# Integrating and Disseminating Pre-Exposure Prophylaxis (PrEP) Screening and Dispensing for Black Men Who Have Sex With Men in Atlanta, Georgia: Protocol for Community Pharmacies

**DOI:** 10.2196/35590

**Published:** 2022-02-09

**Authors:** Natalie D Crawford, Kristin R V Harrington, Daniel I Alohan, Patrick S Sullivan, David P Holland, Donald G Klepser, Alvan Quamina, Aaron J Siegler, Henry N Young

**Affiliations:** 1 Department of Behavioral, Social, and Health Education Sciences Rollins School of Public Health Emory University Atlanta, GA United States; 2 Department of Epidemiology Rollins School of Public Health Emory University Atlanta, GA United States; 3 Division of Infectious Diseases Department of Medicine Emory University School of Medicine Atlanta, GA United States; 4 Division of Medical and Preventative Services Fulton County Board of Health Atlanta, GA United States; 5 Department of Pharmacy Practice and Science College of Pharmacy University of Nebraska Medical Center Omaha, NE United States; 6 National AIDS Education and Services for Minorities Inc Atlanta, GA United States; 7 Department of Clinical and Administrative Pharmacy University of Georgia College of Pharmacy Athens, GA United States

**Keywords:** PrEP, MSM, HIV, prevention, pharmacy, implementation, pre-exposure prophylaxis, men who have sex with men, protocol, integration, dissemination, prophylaxis, screening, race, demographic, sex, sexuality, development, access

## Abstract

**Background:**

Black men who have sex with men (BMSM) suffer from alarmingly high rates of HIV in the United States. Pre-exposure prophylaxis (PrEP) can reduce the risk of HIV infection by 99% among men who have sex with men, yet profound racial disparities in the uptake of PrEP persist. Low PrEP uptake in BMSM is driven by poor access to PrEP, including inconvenient locations of PrEP-prescribing physicians, distrust of physicians, and stigma, which limit communication about PrEP and its side effects. Previous work indicates that offering HIV prevention services in pharmacies located in low-income, underserved neighborhoods is feasible and can reduce stigma because pharmacies offer a host of less stigmatized health services (eg, vaccinations). We present a protocol for a pharmacy PrEP model that seeks to address challenges and barriers to pharmacy-based PrEP specifically for BMSM.

**Objective:**

We aim to develop a sustainable pharmacy PrEP delivery model for BMSM that can be implemented to increase PrEP access in low-income, underserved neighborhoods.

**Methods:**

This study design is a pilot intervention to test a pharmacy PrEP delivery model among pharmacy staff and BMSM. We will examine the PrEP delivery model’s feasibility, acceptability, and safety and gather early evidence of its impact and cost with respect to PrEP uptake. A mixed-methods approach will be performed, including three study phases: (1) a completed formative phase with qualitative interviews from key stakeholders; (2) a completed transitional pilot phase to assess customer eligibility and willingness to receive PrEP in pharmacies during COVID-19; and (3) a planned pilot intervention phase which will test the delivery model in 2 Atlanta pharmacies in low-income, underserved neighborhoods.

**Results:**

Data from the formative phase showed strong support of pharmacy-based PrEP delivery among BMSM, pharmacists, and pharmacy staff. Important factors were identified to facilitate the implementation of PrEP screening and dissemination in pharmacies. During the transitional pilot phase, we identified 81 individuals who would have been eligible for the pilot phase.

**Conclusions:**

Pharmacies have proven to be a feasible source for offering PrEP for White men who have sex with men but have failed to reach the most at-risk, vulnerable population (ie, BMSM). Increasing PrEP access and uptake will reduce HIV incidence and racial inequities in HIV. Translational studies are required to build further evidence and scale pharmacy-based PrEP services specifically for populations that are disconnected from HIV prevention resources.

**International Registered Report Identifier (IRRID):**

DERR1-10.2196/35590

## Introduction

Black men who have sex with men (BMSM) bear the highest burden of HIV in the US*.* Between 2015 and 2019, HIV diagnoses decreased by 18% for White men who have sex with men (WMSM) but decreased only by 5% for BMSM [1,2]. In Georgia—where the proposed study will take place—Black individuals make up less than 40% of the population but represent 71% of new HIV diagnoses [3]. Pre-exposure prophylaxis (PrEP) is the single most effective HIV prevention strategy, yet it is underutilized among BMSM. When taken every day, PrEP reduces the risk of HIV infection by approximately 99% among MSM, yet profound racial disparities in the uptake of PrEP persist [4-6]. Studies estimate that 48-70% of BMSM are willing to use PrEP [7,8]. Yet, uptake among Black individuals is only about 10% [9]. Lower insurance rates among Black compared to White individuals [10] may be a barrier to PrEP uptake. But, evidence of comparable insurance rates among BMSM and WMSM [11], federal legislation mandating PrEP coverage by insurance companies, and supplementary PrEP prescription payment programs do not completely explain significant inequities in PrEP uptake [12]. Indeed, limited access to PrEP, including inconvenient locations of PrEP-prescribing physicians [8,13-17], distrust of physicians, and stigma, which limit communication about PrEP and its side effects [13,17], are noted as critical barriers to PrEP that must be improved to reduce HIV [7,8].

There is a strong scientific premise for increasing PrEP delivery in pharmacies to improve PrEP uptake among BMSM. About 95% of Americans live within 5 miles of a pharmacy; pharmacies have flexible hours, and pharmacists have high credibility with community members [18]. Studies have shown pharmacies can engage with high-risk populations to reduce HIV risk behaviors [19-22] and provide primary prevention services, including immunizations [23], blood pressure screenings [24], and HIV testing [25-28]. PrEP has also been sustainably offered in some pharmacies. In one Seattle pharmacy, almost 100% of mostly WMSM patients initiated PrEP, and 75% followed up for continued PrEP [5]. Following this, 188 Walgreens across the US have offered PrEP through their existing in-pharmacy clinics [29,30]. Existing pharmacy PrEP models, however, have not been tailored for BMSM and are critically needed to reduce HIV transmission and ultimately end the HIV epidemic [31]. Prior work indicates that offering HIV prevention services in pharmacies located in low-income, underserved neighborhoods is feasible [25] and can reduce stigma because pharmacies offer a host of less stigmatized health services (eg, vaccinations) [29]. Further, both MSM and pharmacists have expressed strong support for PrEP screening and dispensing via pharmacies, given their convenience and accessibility [32]. Thus, pharmacy-based HIV prevention services could help overcome stigma and access barriers such as travel.

This protocol describes the development of a culturally appropriate pharmacy PrEP delivery model for BMSM who live in low-income, underserved neighborhoods. This study aims to (1) develop a pharmacy PrEP delivery model by evaluating the barriers to and facilitators of integrating PrEP into existing pharmacy practice among key stakeholders (eg, pharmacists, technicians, PrEP-prescribing physicians, and BMSM); (2) evaluate eligibility and willingness to receive PrEP in pharmacies among BMSM and collect data on customer engagement with PrEP delivery information from pharmacy staff during COVID-19; and (3) pilot test the pharmacy PrEP delivery model and examine its feasibility, acceptability, and safety with respect to PrEP uptake at baseline and at 3-months (the clinically suggested follow-up period) among BMSM.

## Methods

### Design and Evaluation

This pilot intervention tests a pharmacy PrEP delivery model among pharmacy staff and BMSM. We will examine the PrEP delivery model’s feasibility, acceptability, and safety. A mixed-methods approach will be performed, including three study phases: (1) a completed formative phase with qualitative interviews from 30 key stakeholders including BMSM, pharmacists and pharmacy technicians, and PrEP prescribing clinicians; (2) a completed transitional pilot phase to assess customer eligibility and willingness to receive PrEP in pharmacies during COVID-19; and (3) a planned pilot intervention phase which will test delivery of a pharmacy-based PrEP model provided to 60 BMSM in 2 pharmacies in low-income, underserved neighborhoods. We will describe the recruitment, data collection, and planned analyses for each phase.

### Ethics Approval

Ethics approval was obtained from the Emory University Institutional Review Board (IRB00106370).

### Recruitment

#### Formative Phase

A detailed description of the recruitment strategy can be found elsewhere [32]. In brief, participant recruitment during the formative phase varied by key informant group. For pharmacists (n=10) and technicians (n=10), all pharmacies in the highest HIV zip code in Atlanta, as shown by AIDSVu, were identified. AIDSVu is an online, interactive display of HIV prevalence across the US [33]. Using an existing list of all pharmacies in the state of Georgia obtained from the Georgia Board of Pharmacy, we generated a list of pharmacies from high HIV zip codes and then randomly called each pharmacy to determine whether pharmacists or technicians were interested in participating in the study. When we were unable to reach a pharmacy staff person via phone, we visited the pharmacy during normal business hours.

To recruit BMSM, we relied on referrals from our community partners who advertised our study during business hours for MSM seeking HIV prevention services. We also attended weekly social events hosted by our community partner for recruitment. Participants were eligible to participate if they were 18 years of age or older and identified as gay, bisexual, or same-gender-loving.

#### Transitional Pilot Phase

We enrolled 2 pharmacies located in low-income, underserved Atlanta neighborhoods. We obtained consent from at least 1 pharmacist and 2 to 3 technicians at each pharmacy to participate in the study (n=4 per pharmacy).

**Figure 1:**
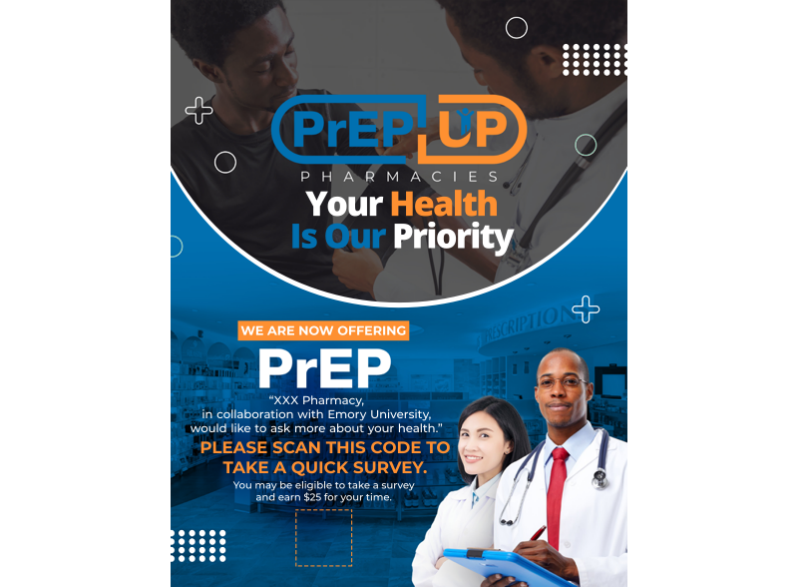
Sample flyers and posters used for participant recruitment. PrEP: pre-exposure prophylaxis.

Posters and flyers were placed in the pharmacy advertising a study about their health to enroll pharmacy customers (Figure 1). Pharmacy staff also placed flyers in customers’ medication delivery packages. The posters and flyers included a QR code that directed customers to an online consent and a 10-question screener survey. Customers who completed the eligibility screener received a US $1 pharmacy coupon regardless of their eligibility in the social behavioral survey. Customers were eligible for the social and behavioral survey if they were: (1) male or trans male, and (2) had any type of sex in the past 6 months, (3) engaged in unprotective sex in the past 6 months, (4) had protected and/or unprotected sex with HIV positive partners in the past 6 months, or (5) injected any drugs in the past 6 months. Eligible customers who completed the social and behavioral survey were compensated US $25 for their time.

#### Pilot Phase

Two pharmacies from the final sampling frame developed during the formative phase will be recruited for the pilot intervention. Eligible pharmacies must have at least two pharmacy staff that are willing to perform study activities which include pharmacy training on the study protocol and methods, informing male customers about the study, and communicating with study staff during the customer recruitment period. Pharmacy customers will be considered eligible and offered pharmacy PrEP access if they are: (1) male or trans male, and (2) had any type of sex in the past 6 months, (3) engaged in unprotective sex in the past 6 months, (4) had protected or unprotected sex with HIV positive partners in the past 6 months, or (5) injected any drugs in the past 6 months. Although this PrEP delivery model is being developed for BMSM, Black race is not included in the eligibility criteria to avoid profiling and potentially stigmatizing one racial group. However, since the pharmacies selected to be a part of the study are located in underserved neighborhoods, we anticipate that most customers will be racial minorities.

### Data Collection

#### Formative Phase

**Figure 2:**
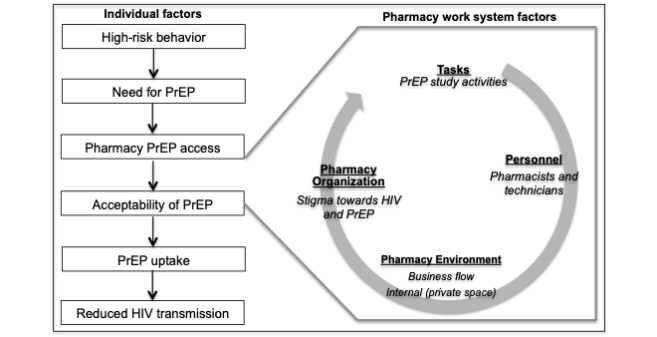
Conceptual framework integrating pharmacy work system influencing PrEP. PrEP: pre-exposure prophylaxis.

Measures included in the study will be guided by the systems engineering initiative for patient safety (SEIPS) multilevel model [34] in order to evaluate whether pharmacy PrEP delivery is organizationally feasible and compatible with pharmacy systems. The conceptual application of SEIPS on pharmacy-based PrEP delivery is shown in Figure 2. The first line of entry to improving pharmacy-based PrEP access relies on implementable study activities. In turn, study activities are completed by the pharmacists and technicians with support from distal organizational structures that enable or prohibit tasks in the pharmacy environment relating to workflow or space. These include but are not limited to time constraints, compensation models, and the ability to complete the required activities for both BMSM and pharmacy staff. Given that pharmacy organizational factors may also underpin stigma towards HIV and HIV prevention services, stigma perceptions among each key stakeholder as well as cultural stigma within the pharmacy organization will also be assessed. Specific questions for pharmacy staff included: support for PrEP screening; policies influencing PrEP delivery; referral and monitoring in pharmacies; potential barriers to the delivery of an intervention in a pharmacy; knowledge of epidemiologic data on HIV; sexual behavior; drug use and PrEP; and sustainability through pharmacy-physician collaboration. In the interviews, we described a preliminary pharmacy-based PrEP screening and delivery model for specific feedback on each step with respect to advertising, engagement of BMSM, costs, and other concerns.

For pharmacists and technicians, we also performed direct observation of the pharmacy to understand routine and nonroutine activities. The door-to-door patient experience of obtaining a prescription and the process implemented behind the counter for filling a prescription were characterized using workflow diagrams for the pharmacy. Patterns were examined across the pharmacy environment to create an overall description that best represents a typical prescription dispensing encounter [35].

#### Transitional Pilot Phase

Two pharmacies were chosen to estimate how many customers would be eligible for the pilot phase. Among those who would be eligible, we assessed their willingness to receive PrEP in pharmacies through an electronic social and behavioral survey. The 20-minute social and behavioral survey included questions on demographics, sex and drug use risk behaviors, mental health, social exposures, and the feasibility, acceptability, and safety of the intervention.

#### Pilot Phase

**Figure 3:**
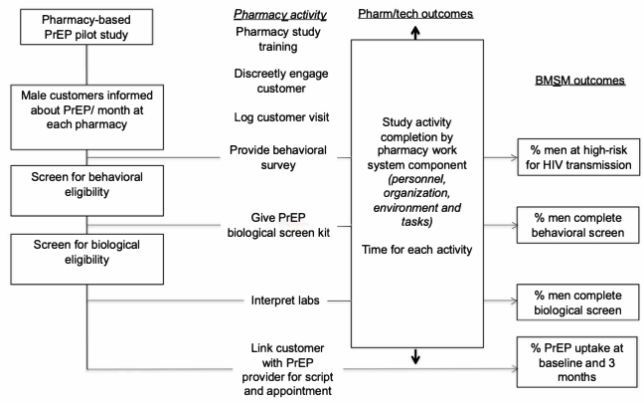
Pilot study activities flow chart. BMSM: Black men who have sex with men; PrEP: pre-exposure prophylaxis.

Pharmacy staff will discreetly inform men (n=60) about the study and determine their willingness to complete an electronic 20-minute social and behavioral screener immediately or schedule an appointment to do so (Figure 3). Pharmacy staff will keep a record of those they approach about the study using an electronic log. Men who agree to participate will be directed to an area where they can privately provide electronic informed consent and complete the survey. If they are willing to participate at a later time, they will be scheduled, and their customer visit will be logged. To circumvent potential literacy issues, the participant will listen to questions via headphones and mark their answers using the touchscreen computer.

The survey will ascertain demographics, sex and drug use risk behaviors, mental health, social exposures, and the feasibility, acceptability, and safety of the intervention. Individuals who report behavioral eligibility for PrEP according to Centers for Disease Control and Prevention guidelines [6] will be offered the opportunity to participate in clinical screening tests by pharmacy staff. The CDC defines PrEP behaviorally appropriate individuals as those who report: (1) having an HIV positive partner or (2) being a gay or bisexual male who engages in unprotected anal intercourse or has had a sexually transmitted infection (STI) in the past 6 months or (3) being a heterosexual male who engages in unprotected intercourse with partners of unknown HIV status who are at substantial risk of HIV infection [6]. We will program the surveys to automatically identify individuals who are behaviorally appropriate for PrEP, and at the end of each survey, a password-protected document will be automatically created that informs the pharmacists or technician if the individual is eligible for the clinical PrEP screening. The pharmacist/technician will only have information on whether the person is eligible or not. They will not know the specific reason that person is eligible as this information will be included in the individuals’ specific data that the pharmacist/technician does not have access to.

Men who are behaviorally eligible for PrEP and agree to the clinical screening tests will be provided with a prepackaged kit of self-administered tests for HIV, rectal chlamydia, syphilis, and gonorrhea and directed back to the private area of the pharmacy to perform their screenings. Video and visual instructions will be provided on a tablet and paper to guide men through the process of each test. Men will also be able to reach a study staff member via phone or videoconference for questions about specimen collection. Those who are not comfortable being screened in the pharmacy will be counseled on the importance of PrEP and HIV prevention and will be given a referral to our community partner. The pharmacist or technician will interpret the results of a rapid HIV OraQuick, rapid rectal chlamydia, syphilis, and gonorrhea tests. Given the sensitivity of the HIV-testing results, the pharmacist will examine those results first in case an individual tests positive. Pharmacy technicians will be trained to deliver HIV negative results, STI results, and creatinine results. The pharmacy will be provided with a documentation system for maintaining the participants' results in a locked file cabinet. Individuals who have an inconclusive HIV test will be told that their test is inconclusive and will be referred to our community partner for a confirmatory HIV test. If a customer tests positive for HIV during the PrEP screening, he will be immediately sent for confirmatory testing and linkage to treatment. Pharmacists will dispense a 7-day starter PrEP prescription for customers who test negative for HIV. They will also be given an appointment with a PrEP-prescribing clinician within the 7-day period. We will complete a 3-month follow-up phone call with men prescribed PrEP to determine whether they continued PrEP use.

### Planned Analyses

#### Formative Phase

All interviews were audio-recorded for verbatim transcription and data analysis. Audio data was immediately transferred to a secure server following every interview. Interviews were transcribed by research assistants with masters-level training in public health and qualitative methods. The research team used a thematic approach [36] using code lists developed inductively from the literature and deductively from the research objective. The code lists were organized by listing codes, definitions, and example quotes from the transcripts. To enhance intercoder reliability, 2 researchers recoded the same transcripts, refined the code lists, and recoded the transcripts. Once coded, all texts were reviewed to conceptualize the inter-relationship between themes and how they related to the research questions. The research team used MAXQDA Analytics Pro (VERBI Software) for data analysis.

#### Transitional Pilot Phase

Exploratory data analysis (EDA) was conducted through data editing using SAS to (1) describe the population of pharmacy patients screened and eligible for PrEP, and (2) examine correlates of willingness to obtain HIV prevention services in the pharmacy. Differences between two means (or medians) were tested using *t* tests or rank tests, and categorical variables will be compared between groups using chi-square tests.

#### Pilot Phase

Formation of new variables and collapsing variables will be done when exploring the proportion and correlates of feasibility, acceptance, safety, and PrEP use at baseline and at 3-months among BMSM. EDA will be conducted through data editing using SAS. EDA will include calculation of means, medians, percentages, proportions, standard deviations, and skewness/kurtosis as appropriate. If outliers or nonstandard distributions exist, variable transformations or standardized cut-points in the data will guide the recoding of continuous variables. Externally validated standards will be used to recode the data if possible. The influence of outliers will be assessed and medians (or rank tests) used if required. Differences between two means (or medians) will be tested using *t* tests or rank tests, and categorical variables will be compared between groups using chi-square tests, exact tests, and with 95% confidence intervals to guide interpretation. Correlates of interest include health insurance, sexual behavior, substance use, and HIV testing behavior. Other relevant mediators and confounders will include variables such as previous or consistent access to health care. We plan to separately examine the relationship between each confounder and outcome of interest using *t* tests or rank tests on continuous measures and exact tests on categorized values if categorizations are used. Initial unadjusted comparisons will be made using exact tests. If significant bivariate associations are found, we will incorporate these exposures as covariates using linear regression for continuous variables and logistic regression for binary variables where sample size allows.

## Results

### Formative Phase

Textbox 1 describes the major findings of the formative interviews for each key stakeholder group. Overall, BMSM, pharmacists, and pharmacy technicians show strong support for pharmacy PrEP delivery. We found important differences between the pharmacist and technician reports that would impact the integration of PrEP into pharmacies. Specifically, pharmacists strongly suggested pharmacy staff training to better support PrEP screening for and dispensing PrEP to patients at high HIV risk, whereas pharmacy technicians highlighted privacy concerns and community support of a pharmacy-based PrEP model.

In the BMSM interviews, we created vignettes to elicit support for pharmacy-based PrEP and asked open-ended questions. The use of the vignette method facilitated increased discussion of sensitive topics and allowed participants to actively participate and place themselves in the hypothetical scenario, which provided greater depth in their responses. For example, participant responses spoke directly to how they would interact with the pharmacist or pharmacy technician and how the pharmacy’s physical space would impact their comfort and perceived privacy. In fact, participants highlighted additional features that would enhance their experience and comfort in this setting, such as a PrEP expert who was on staff wearing a pin to highlight that they were the person to speak to if a customer needed more information.

During the BMSM interviews, we also pilot tested the self-administration of an open-source, electronic social network data collection software, Network Canvas. We used the think aloud method, which is a robust and flexible research technique used to perform usability testing by gathering qualitative information from participants on their cognitive process while completing the interview. Our results suggest that participants were willing to use Network Canvas and found it to be feasible and generally easy to use. However, the sociogram feature, which captures data on the complete social network, required the most instruction for participants. While participants believed that the design of Network Canvas was easy to understand, they had suggestions for improvement, including more intuitive forward buttons with labels noting which was the next step. They suggested the inclusion of a brief tutorial before allowing participants to complete the social network inventory on their own. They also noted a need for features and tools to be consistent on each data collection page to improve the application’s intuitiveness.

**Data collection from BMSM, pharmacists, and pharmacy technicians. BMSM: Black men who have sex with men; PrEP: pre-exposure prophylaxis.**

BMSM:

- Strong support of pharmacy PrEP delivery model.
- Men frequently made frequent purchases and obtained prescriptions and some health services at pharmacies.
- Some men had pre-existing relationships and trust for pharmacy staff.
- Men found pharmacies to be conveniently located and more accessible in their neighborhoods.
- Privacy, confidentiality, and specialized training in HIV and PrEP were important for being comfortable engaging in HIV prevention services.

Pharmacists:

- Strong support of pharmacy PrEP delivery model.
- High levels of comfort providing HIV prevention services, including HIV and STI testing, were reported.
- Increased training for pharmacists and pharmacy staff is needed to help counsel patients.
- Existing infrastructure (eg, relationships with physicians and HIV community organizations, ability to work through payment programs) would support pharmacy PrEP delivery.
- High willingness to make additional structural changes (eg, signage, electronic tablets, off-site training) to promote PrEP delivery.

Pharmacy technicians:

- Strong support of pharmacy PrEP delivery model.
- Strong willingness to obtain HIV prevention and PrEP training, including HIV testing training.
- Increased training for pharmacists and pharmacy staff is needed to help counsel patients.
- Pharmacy technicians are responsible for a number of tasks that could also support PrEP delivery (ie, provision of health and payment information to the patient, prescription and vaccination preparation, etc).
- There are important limitations on the scope of services technicians are allowed to perform (eg, vaccination administration).

### Transitional Pilot Phase

During the transitional pilot phase, 406 individuals completed a 10-question screening survey. Of those, we identified 81 (19.9%) individuals who would have been eligible for the pilot phase based on engaging in high-risk behaviors that would deem them eligible for PrEP. Among this group, 34 (42%) were eligible based upon reported injection drug use with or without risky sexual behaviors, and 47 (58%) were eligible based upon sex risk behaviors alone (Table 1). Most individuals in the sample (50/81, 63%) made US $20,000 or fewer in the past 12 months. Almost half (35/81, 43%) reported ever being homeless, with 17 (20.9%) individuals reporting being homeless in the past 6 months.

**Table 1:** Socio-demographics of the transitional pilot phase study population by injection drug use with or without risky sexual behaviors.

			Total (N=81)		Injection drug use with or without sex risk (N=34)		Sex risk only (N=47)
Age (years), median (IQR)			31 (28-32)		30 (27-32)		31 (2-33)
**Sex, n (%)**							
	Male	79 (97.5)		32 (94.1)		47 (100.0)	
	Female	2 (2.5)		2 (5.9)		0 (0.0)	
**Race/ethnicity, n (%)**							
	Hispanic, Latino, or Spanish	34 (42.0)		24 (70.6)		10 (21.3)	
	Black or African American	17 (21.0)		12 (35.3)		5 (10.6)	
	White	47 (58.0)		15 (44.1)		32 (68.1)	
**Marital status, n (%)**							
	Single, never married	39 (48.1)		16 (47.1)		23 (48.9)	
	Married, or in a domestic partnership	16 (19.8)		7 (20.6)		9 (19.1)	
	Divorced, separated, or widowed	12 (14.8)		10 (29.4)		2 (4.3)	
	Prefer not to answer	14 (17.3)		1 (2.9)		13 (27.7)	
**Highest level of school completed, n (%)**							
	Less than a college degree	24 (29.6)		15 (44.1)		9 (19.1)	
	College degree or greater	57 (70.4)		19 (55.9)		38 (80.9)	
**Total legal income in past 12 months (USD), n (%)**							
	≤$20,000	50 (63.3)		18 (52.9)		32 (71.1)	
	>$20,000	29 (36.7)		16 (47.1)		13 (28.9)	
**Housing history, n (%)**							
	Ever been homeless	35 (43.2)		13 (38.2)		22 (46.8)	
	Homeless in past 6 months	17 (48.6)		4 (30.8)		13 (59.1)	

### Pilot Phase

No preliminary results are available.

## Discussion

Pharmacies have proven to be a feasible source for offering PrEP for WMSM [5,29] but have failed to reach the most at-risk, vulnerable population—BMSM [5]. Our preliminary data indicate that directing pharmacy-based services to communities with a high baseline HIV prevalence could reach individuals at high risk for HIV transmission. State regulations limit pharmacists’ ability to screen and prescribe PrEP in 21 states, so many pharmacies use nurse practitioners (NPs) to administer PrEP programs [29]. But pharmacies in low-income, underserved neighborhoods often lack NPs. Thus, we have designed a multilevel intervention that does not solely rely on pharmacists or NPs to screen BMSM for PrEP. The pilot test of this model will determine the feasibility and acceptability of these procedures in the pharmacy environment. Our ability to increase PrEP access and uptake with pharmacy-delivered services could substantially reduce HIV incidence and racial inequities in HIV.

This study will shift the current paradigm of HIV prevention service delivery for BMSM in three critical ways. First, the proposed model will be developed such that pharmacy PrEP delivery could be achievable for most pharmacists in the US who currently lack the pharmacy-level resources to screen men for PrEP. Second, the proposed research employs a multilevel approach to pharmacy PrEP delivery that assesses not only the population receiving PrEP (BMSM) but also the pharmacy staff who are critical to disseminating PrEP. Evaluation of this model from the targeted population and pharmacy perspective is critical as many pharmacy-based studies have already shown important effects on HIV prevention outcomes among the targeted population, but none have been sustained beyond the study time frame [5,19,21,22,25-28]. Evaluation of each of the pharmacy factors will be guided by SEIPS, a model that is anchored in the industrial engineering subspecialty of human factors but new to public health and behavioral sciences. Given that the model is designed to optimize the system around a given individual (here, BMSM), it can help to identify key barriers and processes that care impair their ability to access PrEP. Hence, the study findings will provide a much-needed understanding of how to incorporate and sustain expanded services by characterizing specific breaks within the pharmacy work system that promote or weaken pharmacy PrEP delivery. Third, to efficiently screen for PrEP eligibility, we will use HIV and STI self-testing kits in a pharmacy—rather than a clinical or home setting [37,38]. This has the secondary benefit of increasing HIV testing.

We acknowledge that this study is subject to a number of limitations. First, selection bias may occur if a customer refuses to enter the study, potentially limiting the generalizability of the MSM sample. Given that this study’s purpose is to test the feasibility of the intervention, we are not concerned about the lack of generalizability of the sample. However, to roughly assess the potential for this bias for a future efficacy trial, pharmacies will record general demographic data on each customer they engage so that those who refuse materials and study information can be compared to those who enroll in the study. These data will be based on observation of race and age (as opposed to inquiry) so that customers will not be discouraged from pharmacy use. Second, the drawback with targeted neighborhood samples is that they may not be representative of the entire target population (BMSM). Although the study may be limited in external validity, it is a prudent first step in examining this population in terms of determining risk for HIV, willingness to be screened and linked to PrEP, and the feasibility, acceptability, and safety of pharmacies as sites to provide PrEP. Third, social desirability bias may occur in the proposed study, as customer sex and drug risk behaviors will be ascertained through self-report. The use of electronic surveys has been shown to improve validity by minimizing invalid reports, such as socially desirable answers, and by ordering questions in a manner that will aid in recall. Additionally, including logic checks in the survey that prevent a respondent from proceeding on the electronic system if their responses are inconsistent can reduce invalid reporting [39]. Participants who consistently contradict themselves in survey responses will be excluded from these analyses. Fourth, we may have insufficient power to adequately detect differences in our primary outcome, given these differences exist. Due to the lack of published (or preliminary) data available to provide evidence of expanded pharmacy services for BMSM, this study will provide preliminary estimates for future efficacy studies. So, it is critical to note that the main goal of this protocol is to first determine if pharmacies can manage a PrEP delivery system for this vulnerable population. This will provide critical information as to whether or not this program can be scaled up, translated, and sustained at a community level.

The contribution of the proposed research is expected to be a sustainable, pharmacy PrEP delivery model that can be implemented without regulatory barriers to increase PrEP access in low-income, underserved neighborhoods for BMSM who have the highest need. This contribution will be significant because it will lay the foundation for making PrEP available in pharmacies specifically for populations that are disconnected from HIV prevention resources.

## Appendix

Multimedia Appendix 1 Peer review summary from the National Institute of Health.

## Abbreviations

BMSM: Black men who have sex with men
CDC: Centers for Disease Control and Prevention
EDA: exploratory data analysis
MSM: men who have sex with men
NP: nurse practitioners
PrEP: pre-exposure prophylaxis
SEIPS: systems engineering initiative for patient safety
STI: sexually transmitted infections
WMSM: White men who have sex with men
