# Supplementary material for: Integrating and Disseminating Pre-Exposure Prophylaxis (PrEP) Screening and Dispensing for Black Men Who Have Sex With Men in Atlanta, Georgia: Protocol for Community Pharmacies
Source: JMIR Res Protoc. 2022 Feb 9;11(2):e35590. doi: 10.2196/35590 (PMC8867290; doi:10.2196/35590)
Supplement: Multimedia Appendix 1 [file resprot_v11i2e35590_app1.pdf]

SUMMARY STATEMENT
(Privileged Communication)

PROGRAM CONTACT:
Michael Stirratt
240-627-3875
stirrattm@mail.nih.gov

Release Date: 08/04/2018
Revised Date:

Application Number: 1 R34 MH119007-01

Principal Investigator

CRAWFORD, NATALIE D

Applicant Organization: EMORY UNIVERSITY

Review Group: BSPH
Behavioral and Social Science Approaches to Preventing HIV/AIDS Study Section
AIDS - EXP. REV.

Meeting Date: 07/11/2018
Council: OCT 2018
Requested Start: 04/01/2019
RFA/PA: PA18-276
PCC: 9A-ASGA

Project Title: Advancing pre-exposure prophylaxis (PrEP) access in pharmacies to improve PrEP uptake in disadvantaged areas
SRG Action: Impact Score:23 Percentile:8 +
Next Steps: Visit https://grants.nih.gov/grants/next_steps.htm
Human Subjects: 30-Human subjects involved - Certified, no SRG concerns
Animal Subjects: 10-No live vertebrate animals involved for competing appl.
Gender: 1A-Both genders, scientifically acceptable
Minority: 1A-Minorities and non-minorities, scientifically acceptable
Children: 3A-No children included, scientifically acceptable

Project Year	Direct Costs Requested	Estimated Total Cost
1	175,000	284,864
2	175,000	284,864
3	100,000	162,780
TOTAL	450,000	732,508

ADMINISTRATIVE BUDGET NOTE: The budget shown is the requested budget and has not been adjusted to reflect any recommendations made by reviewers. If an award is planned, the costs will be calculated by Institute grants management staff based on the recommendations outlined below in the COMMITTEE BUDGET RECOMMENDATIONS section.

1R34MH119007-01 CRAWFORD, NATALIE

RESUME AND SUMMARY OF DISCUSSION: This applicant seeks to develop a pharmacy PrEP delivery model to serve Black men who have sex with men (BMSM) in very poor, racial minority neighborhoods. Black MSM bear a disproportionate burden of HIV and are less likely to have access to PrEP; the ubiquity of pharmacies makes them an ideal venue for PrEP distribution among this population. This application is very innovative in investigating the feasibility of using pharmacies in racial minority neighborhoods to distribute PrEP; it shows further innovation by testing the use of pharmacy space to perform rectal swabs, and by integrating the Systems Engineering Initiative for Patient Safety (SEIPS) approach to determine the feasibility, acceptability, safety, and cost of PrEP through every phase of the intervention. This application is very strong and its methodology and analysis plans are for the most part rigorous. There were, however, some differences of opinion in the weaknesses raised about the application. Comments were made about the PI's lack of external funding; there were concerns about a lack of details in the approach; a lack of clarity in the selection criteria to be used by the pharmacy technicians to offer PrEP screening; it was also not clear how MSM would be identified and recruited. Nevertheless, a majority of the committee felt that these uncertainties were matters that would be disentangled within the context of an R34 and that elucidating the potential of pharmacies as a venue for PrEP distribution for poor Black MSM in minority neighborhoods could potentially have very high impact.

DESCRIPTION (provided by applicant): Optimizing the reach of pre-exposure prophylaxis (PrEP) access for black men who have sex with men (BMSM) is critical to reduce racial inequities in HIV. The overall objective of this R34 application is to develop a culturally appropriate pharmacy PrEP delivery model and examine its feasibility, acceptability, and safety for BMSM who live in high poverty, racial minority neighborhoods. Cohen’s structural theory supports our central hypothesis that increased availability of PrEP screening and prescribing in neighborhoods where BMSM are most impacted by HIV will facilitate PrEP uptake. Findings from the proposed pilot study will support the long- term goal of this program of research, which is to implement an R01 cluster randomized efficacy trial in high poverty, racial minority neighborhoods to increase PrEP uptake among BMSM and reduce racial inequities in HIV. The aims of this R34 feasibility study are to: Aim 1) develop a pharmacy PrEP delivery model by evaluating the barriers to and facilitators of integrating PrEP into existing pharmacy practice among 40 key stakeholders (pharmacists, technicians, PrEP-prescribing physicians and BMSM) and Aim 2) pilot test the pharmacy PrEP delivery model and examine its feasibility, acceptability and safety, and gather early evidence of its impact and cost with respect to PrEP uptake at baseline and in 3-months among BMSM. To accomplish these aims we will conduct a formative phase of in-depth interviews among key stakeholders to inform the intervention development phase, which will establish a pharmacy PrEP delivery model with formalized input from an existing advisory board to be implemented in the pilot study phase among 2 community pharmacies, where we have existing relationships. Pharmacists (n=2) and technicians (n=6) will be trained using an adapted continuing education certified in-pharmacy HIV prevention training. They will complete semi-structured surveys over time (baseline, 3-month and 6-month) to assess the impact of the pharmacy PrEP intervention on pharmacy environment, personnel, and business flow. Social, behavioral surveys will be completed by 60 BMSM. We will follow behaviorally eligible BMSM, who complete their own biological screening in the pharmacy and receive PrEP (n=20) to determine PrEP uptake at 3-months. Development and refinement of this pharmacy PrEP delivery model is significant because it will lay the foundation for pharmacy-based PrEP access that reaches populations historically disconnected from HIV prevention resources. The innovation of this studies lies in its ability to shift the current paradigm of PrEP service delivery for BMSM by 1) creating a model that is achievable for most pharmacists even if they have limited pharmacy-level resources to screen men for PrEP, 2) employing a multi-level approach that examines the impact of the intervention on pharmacy staff and BMSM and 3) testing the use of self-screening in the pharmacy setting.

PUBLIC HEALTH RELEVANCE: The proposed research will develop a culturally appropriate pharmacy PrEP delivery model and examine its feasibility, acceptability, and safety for black men who have sex with men (BMSM) who live in high poverty, racial minority neighborhoods. Increasing access to PrEP through pharmacies has the potential to increase PrEP uptake among BMSM thereby reducing HIV incidence and racial inequities in HIV.

CRITIQUE 1

Significance: 1
Investigator(s): 1
Innovation: 2
Approach: 2
Environment: 1

Overall Impact: The proposed pilot study aims to determine the capacity of pharmacies in African American neighborhoods to screen, counsel and provide PrEP to Black men who have sex with men (BMSM). The study includes 3 phases – formative research with pharmacists, intervention development, and intervention assessment. Their approach is informed by Systems Engineering Initiative for Patient Safety (SEIPS) which is a multi-level approach to assessing feasibility, acceptability and safety of patient services. This approach is integrated at each phase of the study and involves qualitative, quantitative, cost, and observational data collection methods. This outstanding application has numerous strengths. Pharmacies are well positioned to provide preventive services to diverse populations. In most urban communities (even poor ones) they are available, focused on customer service, and interested in expanding the types of services they provide. Studies in other low-income areas with more controversial issues (e.g., syringe access) have found pharmacies to be appropriate sites for expanded services. The significance of the project is high. BMSM are at elevated risk for HIV transmission, yet PrEP uptake rate remain low in the population (<20%). The use of pharmacies could be one, sustainable way for improving PrEP uptake for this high need population. Further, the study is innovative in its use of the physical space of pharmacies, its application of self-testing for HIV and STIs in this setting, and its use of existing relationships with pharmacies to enhance uptake of PrEP. The investigative team is excellent. They possess expertise and experience in all key areas of the proposed study and have conducted preliminary studies that indicate that their approach is acceptable to BMSM. The overall approach is rigorous for a pilot study. They make use of a CAB to inform all aspects of the study, provide incentives to pharmacists and technicians to complete training, plan to collect both cost and observation data on workflows in the intervention, and have an iterative approach to intervention deployment which should allow them to identify potential barriers to deployment of the intervention. Only minor weaknesses were noted. I think the CAB would be strengthened by the inclusion on 1 or 2 BMSM stakeholders and the selection criteria use by technicians to offer PrEP screening is unclear. However, both of these concerns are easily addressed within the parameters of the proposed research. My overall enthusiasm for this novel, high significance intervention development proposal is very high and greatly outweighs my minor concerns.

1. Significance:

Strengths

- BMSM are among the populations at highest risk for HIV transmission. Efforts to improve enrollment in PrEP are needed for this population.

- Pharmacies have been demonstrated to be an effective means for reaching at-risk populations including women with unplanned or suspected pregnancies, BMSM and people who inject drugs.
- Making use of existing commercial enterprises to enhance HIV prevention saves resources and may improve sustainability should interventions prove to be effective.

Weaknesses

- None Noted

2. Investigator(s):

Strengths

- The PI is a new investigator and an assistant professor in the school of public health at Emory University. She has already successfully conducted 3 studies with pharmacies in the area of HIV prevention including HIV testing and providing referral to other indicated preventive services.
- Other investigators have strong complementary skills and backgrounds that support the feasibility of the proposed study.

Weaknesses

- None Noted

3. Innovation:

Strengths

- While pharmacies have been used to provide several types of preventive medications and services, to my knowledge, they have not been used to induce PrEP medication.
- Providing space and materials for self-testing at the pharmacy is innovative.

Weaknesses

- None Noted

4. Approach:

Strengths

- The use of the Systems Engineering Initiative for Patient Safety (SEIPS) approach is a strength.
- The collection of cost data is important and could facilitate adoption of this approach if it is successful and cost are low.
- Methods for the collection of qualitative and quantitative data appear appropriate.
- Preliminary studies indicate that their approach is acceptable to BMSM.
- The use of a CAB is appropriate.
- The approach appears to address potential barriers to pharmacies participating in such a program by providing training and incentives to staff.

Weaknesses

- It was not clear if the CAB included BMSM from the community. If not, a few should be added to the CAB.

- The procedure for selecting pharmacy techs should be clarified.

5. Environment:
Strengths

- The environment is well-prepared to support this research.

Weaknesses

- None Noted

Study Timeline:
Strengths

- None Noted

Weaknesses

- None Noted

Protections for Human Subjects:
Acceptable Risks and/or Adequate Protections

- Procedures for the protection of human subjects are appropriate.

Data and Safety Monitoring Plan (Applicable for Clinical Trials Only):
Not Applicable (No Clinical Trials)

Inclusion of Women, Minorities and Children:

- Sex/Gender: Distribution justified scientifically
- Race/Ethnicity: Distribution justified scientifically
- For NIH-Defined Phase III trials, Plans for valid design and analysis: Not applicable
- Inclusion/Exclusion of Children under 18: Excluding ages <18; justified scientifically
- The sex/gender, race/ethnicity, and children inclusion/exclusion criteria are appropriate.

Vertebrate Animals:
Not Applicable (No Vertebrate Animals)

Biohazards:
Acceptable

Authentication of Key Biological and/or Chemical Resources:
Not Applicable (No Relevant Resources)

Budget and Period of Support:

Recommend as Requested:

CRITIQUE 2

Significance: 5
Investigator(s): 4
Innovation: 4
Approach: 4
Environment: 1

Overall Impact: This R34 application from a junior investigator seeks to develop and pilot a pharmacy-based intervention to increase PrEP access among black MSM (BMSM). The premise is that there are seemingly greater barriers to accessing PrEP among BMSM, and pharmacies represent a more viable option to delivering PrEP than traditional primary care practices. HIV rates are greater among BMSM compared to white MSM, and PrEP uptake is lower. Therefore, increasing uptake rates among BMSM is a priority. The highest risk group for HIV, however, is young BMSM. There is no mention of targeting this intervention for this group, which would dramatically increase the significance of the proposed intervention. The PI does not have a track record of independent funding, and the roles of the Co-I's are not clearly defined in their biosketches. The delivery mechanism (pharmacies) for PrEP is innovative, but the broad focus on all BMSM, versus young BMSM somewhat limits the innovation. Although it is understood that the protocol will be developed and refined based on the qualitative interviews and feedback from participants, there are fundamental questions that if not addressed in the application, call into question the feasibility of the intervention. First, it is not clear who will be approached and how they will be approached. Will all minority men at the pharmacy be asked to participate? How will it be determined that they are MSM? Also, use of pharmacy techs is certainly ideal from a resources and portability standpoint, there are a host of potential issues (comfort level in approaching participants who may be neighbors/friends, being coerced by employer into participating, etc) that were not addressed. The majority of pharmacy staff interviews should be conducted with the techs, not the pharmacists, since they will primarily be in contact with the participants. The environment is excellent.

1. Significance:

Strengths

- HIV rates among black MSM (BMSM) higher than white MSM
- PrEP uptake rates among BMSM lower than white MSM

Weaknesses

- Why is this taking place in a pharmacy? The pharmacist is not involved, and the only requirement is that there is a private room. Did not make the case for a pharmacy vs another, community-based venue.
- Not targeting the highest risk group – young minority MSM. This severely limits the significance of the proposed intervention.

2. Investigator(s):

Strengths

- The study team have participated in several pharmacy-based interventions

Weaknesses

- Dr. Crawford does not have a track record of independent NIH funding
- Not clear the roles of the team from their biosketches. Given the PI is junior, what level of support will she be provided by the team?

3. Innovation:

Strengths

- Delivery of PrEP via pharmacy is potentially innovative.

Weaknesses

- Lack of focus on the most vulnerable population – young minority MSM, lessens the innovation of the study.

4. Approach:

Strengths

- Iterative protocol development
- Preliminary work suggests the intervention may reach target population
- Qualitative data collection to inform intervention development

Weaknesses

- Concern about the techs willingness to participate (interaction with friends/neighbors, coercion by employer)
- How are the men identified?
- Will men really do a rectal swab in the pharmacy room? Highly doubt it.
- One of the issues with accessing PrEP through PCP's is the potential wait time for an appointment. Are there plans in place to mitigate wait times within this particular intervention? What if they only want to see their own physician? This aspect was glossed over.
- Finally, the proposal does not read well. As a result, it is hard to follow.

5. Environment:

Strengths

- Research environment is excellent at Emory

Weaknesses

- None Noted

Study Timeline:

Strengths

- None Noted

Weaknesses

- None Noted

Protections for Human Subjects:
Unacceptable Risks and/or Inadequate Protections

- The potential discomfort of participants conducting their own rectal swabs in a spare room at the neighborhood pharmacy is not addressed.

Data and Safety Monitoring Plan (Applicable for Clinical Trials Only):
Not Applicable (No Clinical Trials)

Inclusion of Women, Minorities and Children:

- Sex/Gender: Distribution justified scientifically
- Race/Ethnicity: Distribution justified scientifically
- For NIH-Defined Phase III trials, Plans for valid design and analysis: Not applicable
- Inclusion/Exclusion of Children under 18: Excluding ages <18; justified scientifically
- Both men and women; all race/ethnicities; 18+

Vertebrate Animals:
Not Applicable (No Vertebrate Animals)

Biohazards:
Not Applicable (No Biohazards)

Authentication of Key Biological and/or Chemical Resources:
Not Applicable

Budget and Period of Support:
Recommend as Requested:

CRITIQUE 3

Significance: 3
Investigator(s): 1
Innovation: 3
Approach: 3
Environment: 1

Overall Impact: This application, submitted by a new investigator proposes to develop a new approach to pharmacy based PrEP distribution for Black Men who have Sex with Men (BMSM), one of the most

vulnerable groups for HIV. The application is very well prepared and the applicant has put together an excellent and comprehensive team of investigators and advisors. The study proposes pharmacist initiated screening and self-testing, and immediate linkage to a prescriber, who will provide a 7 day script. A follow-up appointment will be required to extend the script. The intervention proposed contains many of the issues identified in the proposal about why PrEP use rates are so low in this population, and why they aren't sustained after trials. While I realize that regulations constrain the intervention, it's not clear that the long-term goal of developing a new pharmacy model that does not rely on nurse practitioners can be achieved.

1. Significance:

Strengths

- PrEP is underutilized and initiated late in this key population for the HIV epidemic.
- HIV among BMSM is clustered in poor neighborhoods with low access to HIV care facilities
- Regulatory restrictions inhibit more accessible delivery models, such as community pharmacies, which are ubiquitous in poorer neighborhoods.

Weaknesses

- A number of pharmacy models exist for PrEP

2. Investigator(s):

Strengths

- The PI is Assistant Professor in the Department of Behavioral Sciences at Emory University, and Assistant Director, Prevention Science Core, CFAR, at Emory. She has a 10 year history working on the topic and is well-qualified to serve as PI.
- She is supported by a senior co-I in Patrick Sullivan, PrEP expert staff, as well as experts in the School of Pharmacy at the University of Nebraska.
- A major BMSM AIDS education organization, NAESM, its Director Ernest Walker, and clinical manager Dr. Jamaal Clue will serve as topic experts.
- The proposal adds a very qualified Advisory Board from academia, government and the private sector.

Weaknesses

- Drs. Sullivan, Holland and Siegler provide very little time on the grant, although Dr. Holland will be on-call for the project during regular business hours.

3. Innovation:

Strengths

- Will address issues of pharmacist and pharmacy preparation for PrEP, regulatory issues.
- Focus on sustainability of intervention using a SEIPS (System Engineering Initiative for Patient Safety) process model to identify support and barriers to maintaining the added services.
- The applicant proposes pharmacy self-testing for HIV and STI

Weaknesses

- A number of other pharmacy PrEP programs already exist.

- This model still requires a physician or nurse practitioner on-call. Unclear if this model is sustainable for pharmacies in the poorest neighborhoods.

4. Approach:

Strengths

- Formative research component contains both interview and observation of workflow components. Likely to identify important barriers to implementation and provide important information for intervention development.
- Intervention Development well described, and intervention to be developed closely with participation across a wide range of stakeholders on the investigative team and the advisory board.

Weaknesses

- Physician on-call or nurse practitioner still required. Not sure if intervention proposed is 1) very different from others; and 2) sustainable in this environment for scale-up.

5. Environment:

Strengths

- Emory has excellent resources and the applicant and her team are well placed to take advantage of them.
- Emory has strong links to the communities involved and the key populations.

Weaknesses

- None Noted

Study Timeline:

Strengths

- Comprehensive and integrated

Weaknesses

- None Noted

Protections for Human Subjects:

Acceptable Risks and/or Adequate Protections

- Careful description of processes to maintain confidentiality.

Data and Safety Monitoring Plan (Applicable for Clinical Trials Only):

Acceptable

- No comments

Inclusion of Women, Minorities and Children:

- Sex/Gender: Distribution justified scientifically

- Race/Ethnicity: Distribution justified scientifically
- For NIH-Defined Phase III trials, Plans for valid design and analysis: Not applicable
- Inclusion/Exclusion of Children under 18: Excluding ages <18; justified scientifically
- BMSM are the target population.

Vertebrate Animals:
Not Applicable (No Vertebrate Animals)

Biohazards:
Not Applicable (No Biohazards)

Authentication of Key Biological and/or Chemical Resources:
Not Applicable (No Relevant Resources)

Budget and Period of Support:
Recommend as Requested:

THE FOLLOWING SECTIONS WERE PREPARED BY THE SCIENTIFIC REVIEW OFFICER TO SUMMARIZE THE OUTCOME OF DISCUSSIONS OF THE REVIEW COMMITTEE, OR REVIEWERS' WRITTEN CRITIQUES, ON THE FOLLOWING ISSUES:

PROTECTION OF HUMAN SUBJECTS: ACCEPTABLE

INCLUSION OF WOMEN PLAN (G1A): ACCEPTABLE

INCLUSION OF MINORITIES PLAN (M1A): ACCEPTABLE

INCLUSION OF CHILDREN PLAN (C3A): ACCEPTABLE

COMMITTEE BUDGET RECOMMENDATIONS: The budget was recommended as requested.

Footnotes for 1 R34 MH119007-01; PI Name: CRAWFORD, NATALIE D

+ Derived from the range of percentile values calculated for the study section that reviewed this application.

NIH has modified its policy regarding the receipt of resubmissions (amended applications). See Guide Notice NOT-OD-14-074 at http://grants.nih.gov/grants/guide/notice-files/NOT-OD-14-074.html. The impact/priority score is calculated after discussion of an application by averaging the overall scores (1-9) given by all voting reviewers on the committee and multiplying by 10. The criterion scores are submitted prior to the meeting by the individual reviewers assigned to an application, and are not discussed specifically at the review meeting or calculated into the overall impact score. Some applications also receive a percentile

ranking. For details on the review process, see http://grants.nih.gov/grants/peer_review_process.htm#scoring.

MEETING ROSTER

Behavioral and Social Science Approaches to Preventing HIV/AIDS Study Section
AIDS and Related Research Integrated Review Group
CENTER FOR SCIENTIFIC REVIEW
BSPH
07/11/2018 - 07/12/2018

Notice of NIH Policy to All Applicants: Meeting rosters are provided for information purposes only. Applicant investigators and institutional officials must not communicate directly with study section members about an application before or after the review. Failure to observe this policy will create a serious breach of integrity in the peer review process, and may lead to actions outlined in NOT-OD-14-073 at https://grants.nih.gov/grants/guide/notice-files/NOT-OD-14-073.html and NOT-OD-15-106 at https://grants.nih.gov/grants/guide/notice-files/NOT-OD-15-106.html, including removal of the application from immediate review.

CHAIRPERSON(S)

ALLEN, SUSAN A, MD
PROFESSOR
DEPARTMENT OF PATHOLOGY
AND LABORATORY MEDICINE
SCHOOL OF MEDICINE
EMORY UNIVERSITY
ATLANTA, GA 30322

MEMBERS

BARAL, STEFAN DAVID, MD *
ASSOCIATE PROFESSOR
DEPARTMENT OF EPIDEMIOLOGY
BLOOMBERG SCHOOL OF PUBLIC HEALTH
JOHNS HOPKINS UNIVERSITY
BALTIMORE, MD 21205

BAUERMEISTER, JOSE ARTURO, PHD
PROFESSOR
DEPARTMENT OF FAMILY AND COMMUNITY HEALTH
SCHOOL OF NURSING
UNIVERSITY OF PENNSYLVANIA
PHILADELPHIA, PA 19104

BAUMAN, LAURIE J, PHD
PROFESSOR
DEPARTMENT OF PEDIATRICS
ALBERT EINSTEIN COLLEGE OF MEDICINE
BRONX, NY 10461

BLUTHENTHAL, RICKY N, PHD *
PROFESSOR
DEPARTMENT OF PREVENTIVE MEDICINE
KECK SCHOOL OF MEDICINE
UNIVERSITY OF SOUTHERN CALIFORNIA
LOS ANGELES, CA 90033

BOGART, LAURA M, PHD *
SENIOR BEHAVIORAL SCIENTIST
RAND CORPORATION
SANTA MONICA, CA 90407-2138

BROUWER, KIMBERLY C, PHD *
PROFESSOR
DIVISION OF GLOBAL HEALTH
DEPARTMENT OF MEDICINE
SCHOOL OF MEDICINE
UNIVERSITY OF CALIFORNIA, SAN DIEGO
LA JOLLA, CA 92093

CHARLEBOIS, EDWIN DUNCAN, PHD *
PROFESSOR
DEPARTMENT OF MEDICINE
SCHOOL OF MEDICINE
UNIVERSITY OF CALFORNIA, SAN FRANCISCO
SAN FRANCISCO, CA 94105

DICKSON-GOMEZ, JULIA B, PHD
PROFESSOR
CENTER FOR AIDS INTERVENTION RESEARCH
DEPARTMENT OF PSYCHIATRY AND BEHAVIORAL MEDICINE
MEDICAL COLLEGE OF WISCONSIN
MILWAUKEE, WI 53202

DOMBROWSKI, KIRK, PHD *
PROFESSOR
DEPARTMENT OF SOCIOLOGY
COLLEGE OF ARTS AND SCIENCES
UNIVERSITY OF NEBRASKA-LINCOLN
LINCOLN, NE 68588

HAVENS, JENNIFER R, PHD *
ASSOCIATE PROFESSOR
DEPARTMENT OF BEHAVIORAL SCIENCE
COLLEGE OF MEDICINE
UNIVERSITY OF KENTUCKY
LEXINGTON, KY 40504

HIGHTOW-WEIDMAN, LISA B, MD, MPH
ASSOCIATE PROFESSOR
DIVISION OF INFECTIOUS DISEASES
DEPARTMENT OF MEDICINE
SCHOOL OF MEDICINE
UNIVERSITY OF NORTH CAROLINA
CHAPEL HILL, NC 27599-7030

KENDALL, CARL, PHD *
PROFESSOR
DEPARTMENT OF GLOBAL COMMUNITY HEALTH
AND BEHAVIORAL SCIENCES
SCHOOL OF MEDICINE
TULANE UNIVERSITY
NEW ORLEANS, LA 70112

KERSHAW, TRACE S, PHD
PROFESSOR
CENTER FOR INTERDISCIPLINARY RESEARCH ON AIDS
DEPARTMENT OF EPIDEMIOLOGY
SCHOOL OF PUBLIC HEALTH
YALE UNIVERSITY
NEW HAVEN, CT 06510

KURTZ, STEVEN P, PHD *
PROFESSOR AND DIRECTOR
CENTER FOR APPLIED RESEARCH ON SUBSTANCE USE
AND HEALTH DISPARITIES
DEPARTMENT OF JUSTICE AND HUMAN SERVICES
NOVA SOUTHEASTERN UNIVERSITY
CORAL GABLES, FL 33134

LEWIS, CRYSTAL FULLER, PHD *
ASSOCIATE PROFESSOR
DEPARTMENT OF PSYCHIATRY
LANGONE MEDICAL SCHOOL
NEW YORK UNIVERSITY
NEW YORK, NY 10016

MACQUEEN, KATHLEEN M, PHD *
SENIOR SCIENTIST
SOCIAL AND BEHAVIORAL HEALTH SCIENCES
FHI 360
DURHAM, NC 27701

MEEK, ERIN, DRPH *
SENIOR RESEARCH SCIENTIST
AIDS OFFICE
SAN FRANCISCO DEPARTMENT OF PUBLIC HEALTH
SAN FRANCISCO, CA 94102

MENA, LEANDRO ANTONIO, MD *
PROFESSOR AND CHAIR
POPULATION HEALTH SCIENCE
JOHN D BOWER SCHOOL OF POPULATION HEALTH
UNIVERSITY OF MISSISSIPPI MEDICAL CENTER
JACKSON, MS 39216

PAGE, KIMBERLY, PHD
PROFESSOR AND CHIEF
DIVISION OF EPIDEMIOLOGY, BIOSTATISTICS,
AND PREVENTIVE MEDICINE
DEPARTMENT OF INTERNAL MEDICINE
UNIVERSITY OF NEW MEXICO HEALTH SCIENCES CENTER
ALBUQUERQUE, NM 87131

RAMSEY, SUSAN E, PHD
ASSOCIATE PROFESSOR
DIVISION OF GENERAL INTERNAL MEDICINE
RHODE ISLAND HOSPITAL
PROVIDENCE, RI 02903

ROSENBERG, ELI SAMUEL, PHD *
ASSOCIATE PROFESSOR
DEPARTMENT OF EPIDEMIOLOGY AND BIOSTATISTICS
SCHOOL OF PUBLIC HEALTH
UNIVERSITY AT ALBANY, STATE UNIVERSITY OF NEW YORK
RENSSELAER, NY 12144

ROSSER, B R SIMON, PHD *
PROFESSOR
DIVISION OF EPIDEMIOLOGY AND COMMUNITY HEALTH
SCHOOL OF PUBLIC HEALTH
UNIVERSITY OF MINNESOTA
MINNEAPOLIS, MN 55454

SANDFORT, THEODORUS G M, PHD *
PROFESSOR
DEPARTMENT OF PSYCHIATRY
MAILMAN SCHOOL OF PUBLIC HEALTH
COLUMBIA UNIVERSITY
NEW YORK, NY 10032

SCHNEIDER, JOHN, MD, MPH
ASSOCIATE PROFESSOR
DEPARTMENT OF MEDICINE
UNIVERSITY OF CHICAGO
CHICAGO, IL 60637

SIMONI, JANE MARIE, PHD
PROFESSOR
DEPARTMENT OF PSYCHOLOGY
UNIVERSITY OF WASHINGTON
SEATTLE, WA 98195

SWEAT, MICHAEL D, PHD
PROFESSOR
DEPARTMENT OF PSYCHIATRY
AND BEHAVIORAL SCIENCES
MEDICAL UNIVERSITY OF SOUTH CAROLINA
CHARLESTON, SC 29407

TURAN, JANET M, PHD
PROFESSOR
DEPARTMENT OF HEALTH CARE ORGANIZATION
AND POLICY
SCHOOL OF PUBLIC HEALTH
UNIVERSITY OF ALABAMA AT BIRMINGHAM
BIRMINGHAM, AL 35294

MAIL REVIEWER(S)

DEL VALLE, SARA, PHD
DEPUTY GROUP LEADER
DEFENSE SYSTEMS AND ANALYSIS DIVISION
LOS ALAMOS NATIONAL LABORATORY
LOS ALAMOS, NM 87545

GOODREAU, STEVEN M., PHD
ASSOCIATE PROFESSOR
DEPARTMENT OF ANTHROPOLOGY
UNIVERSITY OF WASHINGTON
SEATTLE, WA 98105

SCIENTIFIC REVIEW OFFICER

GUERRIER, JOSE H, PHD
SCIENTIFIC REVIEW OFFICER
CENTER FOR SCIENTIFIC REVIEW
NATIONAL INSTITUTES OF HEALTH
BETHESDA, MD 20892

EXTRAMURAL SUPPORT ASSISTANT

STROTHERS, DIARA
EXTRAMURAL SUPPORT ASSISTANT
CENTER FOR SCIENTIFIC REVIEW
NATIONAL INSTITUTES OF HEALTH
BETHESDA, MD 20892

* Temporary Member. For grant applications, temporary members may participate in the entire meeting or may review only selected applications as needed.

Consultants are required to absent themselves from the room during the review of any application if their presence would constitute or appear to constitute a conflict of interest.
